# The Seroprevalence of Dengue Virus Infection and Its Association With Iron (Fe) Level in Pregnant Women in Guangzhou, China

**DOI:** 10.3389/fmed.2021.759728

**Published:** 2021-12-10

**Authors:** Jiong Wang, Jiufeng Sun, Limei Sun, Yufeng Ye, Hanwei Chen, Jianpeng Xiao, Guanhao He, Jianxiong Hu, Guimin Chen, He Zhou, Xiaomei Dong, Wenjun Ma, Bo Zhang, Tao Liu

**Affiliations:** ^1^School of Public Health, Southern Medical University, Guangzhou, China; ^2^Guangdong Provincial Institute of Public Health, Guangdong Provincial Center for Disease Control and Prevention, Guangzhou, China; ^3^Guangdong Provincial Center for Disease Control and Prevention, Guangzhou, China; ^4^Guangzhou Panyu Central Hospital, Guangzhou, China; ^5^Department of Public Health and Preventive Medicine, School of Medicine, Jinan University, Guangzhou, China; ^6^Food Safety and Health Research Center, School of Public Health, Southern Medical University, Guangzhou, China

**Keywords:** dengue, iron, trace elements, IgG antibodies, pregnant women

## Abstract

Dengue fever is regarded as the most prevalent mosquito-borne viral disease in humans. However, information of dengue virus (DENV) infection in pregnant women and the influence factors remain unclear. In this study, we extracted information of 2,076 pregnant women from the Prenatal Environment and Offspring Health (PEOH) birth cohort conducted since 2016 in Guangzhou, China. Peripheral blood and clean midstream urine samples of participants were collected during their hospitalization for childbirth. Indirect enzyme-linked immunosorbent assay (ELISA) was used to detect immunoglobulin G (IgG) antibodies of DENV in serum samples, and inductively coupled plasma mass spectrometry (ICP-MS) was applied to determine the Fe concentrations in the urine samples, which were then adjusted for by urine creatinine and transformed by natural logarithm (ln-Fe). The seroprevalence of DENV IgG antibody in all included participants was 2.22% (46/2,076). We observed higher seroprevalence of IgG antibody in women aged ≥35 years (2.9%), education ≤ 12 years (2.5%), yearly income per capita <100,000 yuan (2.4%), no use of air-conditioner (2.4%), no use of mosquito coils (2.3%), and no exercise during pregnancy (4.1%). A U-shaped relationship was found between ln-Fe concentration and the risk of positive IgG antibody. Compared with women with ln-Fe concentration of 2.0–2.9 μg/g creatinine, slightly higher risks of positive IgG antibody were found among women with ≤2.0 (RR = 4.16, 95% CI: 0.78, 19.91), 3.0–3.9 (RR = 1.93, 95% CI: 0.65, 7.08), 4.0–4.9 (RR = 2.19, 95% CI: 0.65, 8.51), and ≥5.0 μg/g creatinine of ln-Fe (RR = 2.42, 95% CI: 0.46, 11.33). Our findings suggested that the seroprevalence of dengue IgG antibody in pregnant women was comparable to the general population in Guangzhou, China. The risk of DENV infection may be associated with maternal demographic characteristics and behaviors. Both maternal low and high Fe concentrations may be positively associated with the risk of DENV infection.

## Introduction

Dengue is an arthropod-borne infectious disease caused by dengue virus (DENV) infection and transmitted by Aedes mosquitoes (1). It is regarded as the fastest spreading mosquito-borne viral disease, and the global incidence has increased 30 times in the past half century (2). World Health Organization (WHO) estimated in 2012 that 50–100 million new infections annually occurred in 100 endemic countries, which has resulted in a heavy economic burden worldwide (2). In China, the scope and regions affected by DENV have gradually expanded from Hainan and Guangdong provinces to Fujian, Zhejiang, Henan, and other northern regions (3–6). Dengue fever has become a vector-borne disease that needs to be controlled in priority in China.

It is worth noting that pregnant woman is a susceptible group in dengue-endemic areas. Evidence from India, Malaysia, and Brazil showed that the positive rate of serum DENV immunoglobulin G (IgG) antibodies in pregnant women were as high as 30.41, 32.4, and 53.9%, respectively (7–9). After infection of DENV, a higher risk of severe dengue fever was observed in pregnant women than in other groups of people (10). Studies have also suggested that DENV infection during pregnancy was linked to pregnant complications such as bleeding and cesarean delivery, adverse pregnant outcomes such as low birth weight, premature delivery, miscarriage, and stillbirth, and even maternal death (11–14). Moreover, although dengue is a mosquito-borne disease, many studies have reported vertical transmission from mother to neonate (15–17).

The risk factors of DENV infection and epidemic remain unclear, and may be related to weather, mosquito vectors, and social factors (18–22). Recent studies suggested that the infection and prognosis of dengue fever may be related to trace elements (23, 24). For example, an animal experimental study reported that in the mouse-mosquito infection model, Aedes aegypti infection with dengue fever is negatively correlated with the serum iron (Fe) concentrations of blood donors, which indicates that Fe deficiency in the population may promote the transmission of DENV through mosquito vectors (25). However, there is still a lack of evidence from epidemiological studies. Therefore, conducting epidemiological studies to explore the relationship between dengue infection and Fe level in body may be of great significance for preventing dengue infection and improving prognosis of pregnant women.

Guangdong Province is located in South China covered by a subtropical climate, and is the major epidemic area of dengue fever in China (26, 27). For example, an unprecedented dengue fever outbreak occurred in Guangdong Province in 2014, and a total of 45,224 dengue fever cases were reported, which accounted for about 70% of the total reported cases in China in 2014 (28). Although several studies have investigated the prevalence of DENV infection using IgG antibody in the general population (29–32), only one study was found to investigate the infection of DENV in pregnant women (33). Therefore, more research works are needed to investigate the prevalence of DENV infection and to explore the impact factors in pregnant women. Such studies could provide valuable information for clinical workers to understand the health effects of DENV infection on pregnant women, and take measures to prevent the complications and adverse pregnant outcomes.

To fill in above research gaps, this study used information of 2,076 pregnant women from the Prenatal Environment and Offspring Health (PEOH) birth cohort conducted since 2016 in Guangzhou, China. We aimed to investigate the seroprevalence of DENV infection in pregnant women, and to estimate the associations of iron exposure levels with DENV infection risk.

## Materials and Methods

### Study Subjects

Information of all study subjects were extracted from the PEOH birth cohort study conducted since 2016 in Guangzhou, China, which has been described elsewhere (34–36). Briefly, pregnant women were initially recruited from the antenatal care outpatient unit of Panyu Central Hospital which is the largest regional hospital. The inclusion criteria were: (1) in the early pregnancy (gestational weeks ≤ 13 weeks); (2) aged 18–50 years old; (3) with no severe diseases including hyperthyroidism, heart disease, chronic kidney disease, tuberculosis, and psychiatric disease. In contrast, the exclusion criteria were: (1) with occupational exposure to Fe-related works; (2) refuse to answer the questionnaire; (3) having difficulties in communication.

### Data Collection

Each recruited pregnant woman was interviewed face-to-face using a questionnaire by trained public health professionals to collect their information including maternal demographic characteristics, life behaviors, household circumstances, and diets. Other information such as weight and height were extracted from maternal medical records. This information were recorded as baseline data, and a follow-up profile was set up for each participant. A follow-up investigation was conducted for study subjects during their hospitalization for childbirth (late pregnancy), during which another round of face-to-face questionnaire investigation was conducted, and each participant has collected 5.0 ml peripheric venous blood and 15.0 ml of clean midstream urine during 24 h after hospitalization. The second survey mainly collected their information after the baseline investigation. The baseline investigation was started in June 2016, and all follow-up investigations were finished in December 2017.

**Figure 1:**
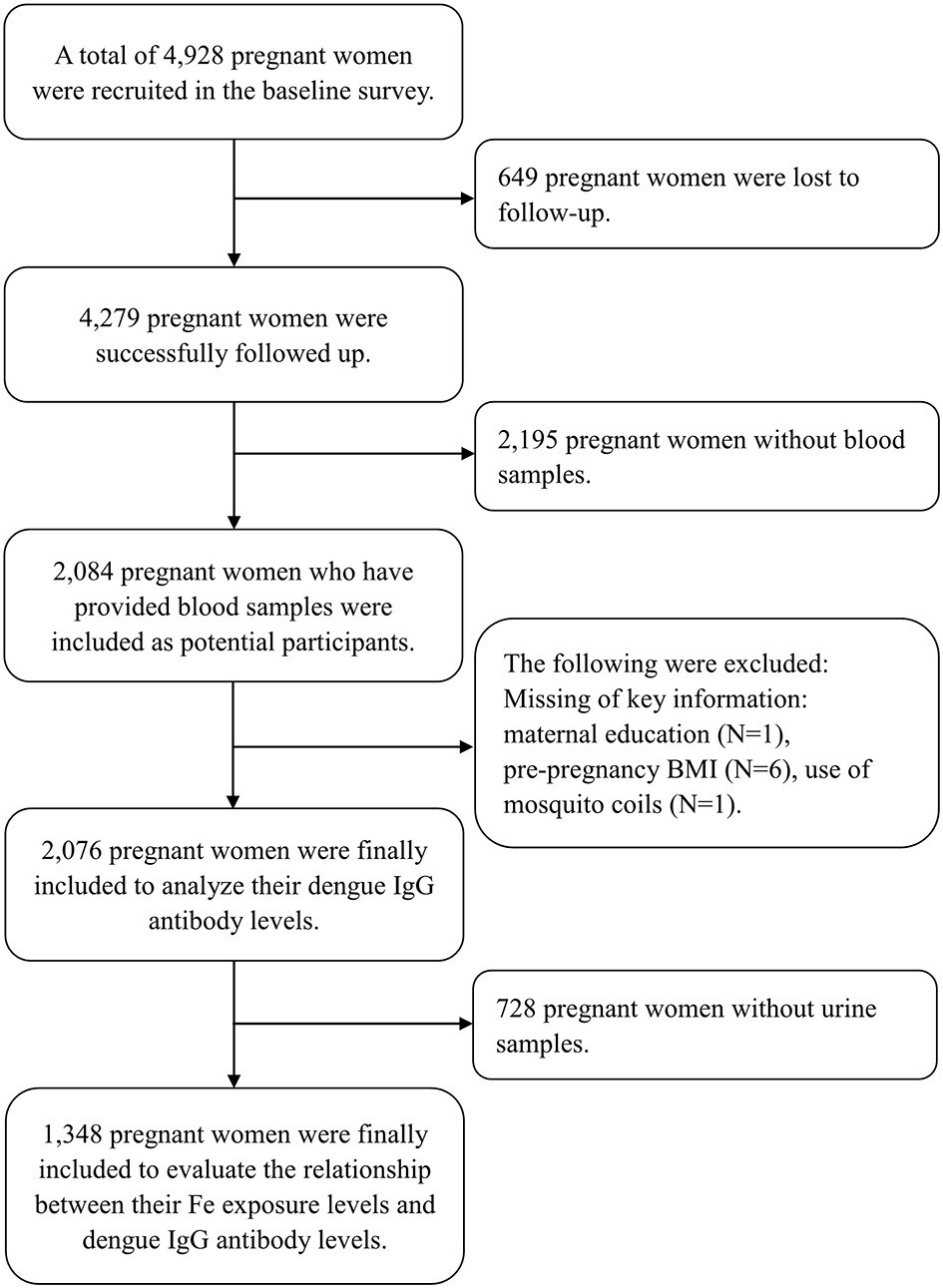
Chart of the cohort study participants selection.

A total of 4,928 pregnant women were initially recruited in the baseline investigation, and 4,279 were successfully followed up (86.8%), out of whom 2,084 pregnant women whose blood samples were collected were included as potential participants. We further excluded participants lacking key variables, such as maternal education (*N* = 1), pre-pregnancy BMI (body mass index, kg/m^2^) (*N* = 6), and use of mosquito coils (*N* = 1). Finally, 2,076 pregnant women were included to depict the seroprevalence of DENV infection and to explore its influence factors (Figure 1). Among the 2,076 pregnant women, we further excluded participants who did not provide the urine sample (*N* = 728). A total of 1,348 pregnant women were finally included to estimate the association of maternal Fe level with dengue infection (Figure 1).

### Detection of DENV IgG Antibody

All blood samples were immediately refrigerated and transferred to the center laboratory after collection, and were stored at −80°C for later laboratory detection. We used the Panbio^®^ Dengue IgG Indirect ELISA kits (Panbio, Australia, Reagent Lot Number: 01P30F006) to detect the IgG antibody level of DENV in the serum samples of pregnant women according to the kit instructions. The specificity of the ELISA was 99.2% and the sensitivity was 92.5% (37). Samples with Panbio units >11 were considered as positive, <9 were considered as negative, and repeated experiments were conducted to test samples with Panbio units between 9 and 11 (38, 39), and if the result is still between 9 and 11, for the sake of conservatism, we defined it to be negative.

### Determinations of the Urinary Concentrations of Fe and Creatinine

We collected 15.0 ml of clean midstream urine samples of subjects during their hospitalization for childbirth (late pregnancy) and stored them in a polypropylene tube for refrigeration, immediately transferred to the central laboratory and stored at −80°C for later laboratory detection. The methods of measuring the concentrations of metals and creatinine have been described in detail elsewhere (36). Briefly, after completely melted at room temperature, the urine samples were tested using inductively coupled plasma mass spectrometer (ICP-MS) (Agilent 7700x, Agilent Technologies). The measurement conditions of ICP-MS included 1,550 W of radio frequency power, 15.00 L/min of plasma gas flow, 1.14 L/min of carrier gas flow, and 4.5 L/min of helium gas flow. The limit of detection (LOD) for Fe was 0.01 mg/L. Samples with a concentration below the LOD were imputed as half of the LOD. Among the 1,348 samples, 525 samples had Fe concentrations below the LOD. All the original Fe concentration was calibrated by the corresponding urinary creatinine concentration, which was detected by an automatic biochemical analyzer (Hitachi 7600-020) (40). The creatinine-corrected Fe concentrations (CC-Fe) were then transformed by the natural logarithm (ln-Fe, μg/g creatinine) because their distribution was right-skewed.

### Statistical Analysis

Categorial variables were expressed as N (%), and continuous variables were expressed as mean ± standard deviation (SD). A logistic regression model was used to estimate the crude and adjusted associations of positive dengue IgG antibody with variables including demographic characteristics, life behaviors, household circumstances, and diets. A generalized linear model (GLM) was used to estimate the crude and adjusted relationships between maternal urine ln-Fe levels and positive dengue IgG antibody. In the multivariate GLM model, the following variables were adjusted for as potential confounders: maternal age, education, occupation, yearly income per capita, pre-pregnancy BMI, air-conditioner use, use of mosquito coils, exercise, consumptions of vitamin, and vegetables. These confounders were selected based on literature review and definition of a cofounder. The urine Ln-Fe levels were input to the GLM model as continuous variable and categorial variables divided into five groups [Group 1 (≤2.0 μg/g creatinine), Group 2 (2.0~2.9 μg/g creatinine), Group 3 (3.0~3.9 μg/g creatinine), Group 4 (4.0~4.9 μg/g creatinine), and Group 5 (≥5.0 μg/g creatinine)]. We reported the relative risk (RR) and its 95% confidence interval (CI) in above logistic regression and GLM model analyses. All analyses were performed using R3.6.1 (R Development Core Team 2019, https://www.r-project.org). All tests were two-sided, and *P* < 0.05 was considered statistically significant.

### Ethics Statement

The Ethics Committee of the Review Board of Guangdong Provincial Center for Disease Control and Prevention approved the protocol of this study, and the research has been registered in the Chinese Clinical Trial Registry (CHICTR-ROC-17013496). Each recruited participant signed a written informed consent form. The data used in this study were anonymous and without identifiable private information.

## Results

### General Characteristics of Study Participants

The study finally included 2,076 pregnant women whose blood samples were collected, of which 1,348 provided urine samples. Table 1 describes the general characteristics of all participants. Among the 2,076 participants, 650 (31.3%) aged 35 years or over, 1,219 (58.7%) had an education >12 years, 1,355 (65.3%) had yearly income per capita <100,000 yuan, and 275 (13.2%) were overweight or obesity. Similar distributions of above variables were found in those 1,348 participants who provided the urine samples. The mean concentration of ln-Fe concentration was 3.58 (SD = 1.04) μg/g creatinine. The percentages of pregnant women in the five groups [Group 1 (≤2.0 μg/g creatinine), Group 2 (2.0–2.9 μg/g creatinine), Group 3 (3.0–3.9 μg/g creatinine), Group 4 (4.0–4.9 μg/g creatinine), and Group 5 (≥5.0 μg/g creatinine)] were 4.6, 26.0, 38.2, 21.2, and 10.0%, respectively.

**Table 1:** General characteristics of study participants.

	**Participants who**		**Participants who**	
	**provide**		**provided**	
	**blood samples**		**urine samples**	
	***N***	**%**	***N***	**%**
**Total**	2,076	100.0	1,348	100.0
**Maternal age (years)**				
<35	1,426	68.7	928	68.8
≥35	650	31.3	420	31.2
**Maternal education (years)**				
≤ 12	857	41.3	549	40.7
>12	1,219	58.7	799	59.3
**Yearly income per capita (×1,000 Yuan)**				
<100	1,355	65.3	852	63.2
≥100	697	33.6	482	35.8
Refused to answer/Missing	24	1.1	14	1.0
**Pre-pregnancy BMI (kg/m** ^**2**^ **)**				
<24.0	1,801	86.8	1,176	87.2
≥24.0 (Overweight or Obesity)	275	13.2	172	12.8
**Maternal occupation**				
Manual worker	113	5.4	70	5.2
Housewife	206	9.9	138	10.2
Technician	371	17.9	265	19.7
Business	1,115	53.7	707	52.4
Unemployment	163	7.9	109	8.1
Others	108	5.2	59	4.4
**Air-conditioner use**				
No	169	8.1	112	8.3
Yes	1,907	91.9	1,236	91.7
**Use of mosquito coils**				
No	1,873	90.2	1,226	90.9
Yes	203	9.8	122	9.1
**Exercise**				
No	220	10.6	121	9.0
Yes	1,856	89.4	1,227	91.0
**Vitamin consumption (times/week)**				
<5	758	36.5	502	37.2
≥5	1,237	59.6	794	58.9
Refused to answer/Missing	81	3.9	52	3.9
**Vegetable consumption (times/week)**				
≤ 7	202	9.7	110	8.1
8~14	734	35.4	505	37.5
≥15	1,100	53.0	708	52.5
Refused to answer/Missing	40	1.9	25	1.9
**ln-Fe (μg/g creatinine)**				
Group 1 (≤ 2.0)	–	–	62	4.6
Group 2 (2.0~)	–	–	351	26.0
Group 3 (3.0~)	–	–	514	38.2
Group 4 (4.0~)	–	–	286	21.2
Group 5 (≥5.0)	–	–	135	10.0

*BMI, body mass index; Ln-Fe: the urinary Fe concentrations were corrected by creatinine and transformed by natural logarithm; –, Not applicable*.

### Seroprevalence of DENV IgG Antibody and Its Influence Factors

Out of the total 2,076 pregnant women, 46 had a positive dengue IgG antibody-positive blood samples, with a positive rate of 2.22% (46/2,076). Table 2 summarized the positive rates of serum IgG antibodies in different groups of pregnant women. We observed higher prevalence of IgG antibody in pregnant women with age ≥35 years old (2.9%), education ≤ 12 years (2.5%), yearly income per capita <100,000 yuan (2.4%), pre-pregnancy BMI <24.0 (2.3%), no use of air-conditioner (2.4%), no use of mosquito coils (2.3%), and no exercise during pregnancy (4.1%). In addition, the positive rates of IgG antibodies in manual workers (3.5%), technicians (2.4%), and business personnel (2.4%) were higher than other occupations.

**Table 2:** Variables related to positive IgG antibody of dengue fever (*n* = 2,076).

	**IgG Negative** ***n* (%)**	**IgG Positive** ***n* (%)**	**χ^2^**	***P***	**Crude** **RR (95% CI)**	**Adjusted** **RR (95% CI)^a^**
**Maternal age (years)**						
<35	1,399 (98.1)	27 (1.9)	1.74	0.188	Reference	Reference
≥35	631 (97.1)	19 (2.9)			1.56 (0.85, 2.81)	1.58 (0.85, 2.90)
**Maternal education (years)**						
≤ 12	836 (97.5)	21 (2.5)	0.21	0.647	1.20 (0.66, 2.16)	1.24 (0.63, 2.43)
>12	1,194 (97.9)	25 (2.1)			Reference	Reference
**Yearly income per capita**						
**(×1,000 Yuan)**						
<100	1,323 (97.6)	32 (2.4)	–	0.387^b^	1.27 (0.68, 2.53)	1.30 (0.67, 2.66)
≥100	684 (98.1)	13 (1.9)			Reference	Reference
Refused to answer/Missing	23 (95.8)	1 (4.2)			2.29 (0.12, 12.26)	2.46 (0.13, 13.67)
**Pre-pregnancy BMI (kg/m** ^**2**^ **)**						
<24.0	1,760 (97.7)	41 (2.3)	0.07	0.794	1.26 (0.54, 3.67)	1.47 (0.61, 4.35)
≥24.0 (Overweight or Obesity)	270 (98.2)	5 (1.8)			Reference	Reference
**Maternal occupation**						
Unemployment	161 (98.8)	2 (1.2)			Reference	Reference
Manual worker	109 (96.5)	4 (3.5)			2.95 (0.57, 21.58)	2.20 (0.41, 16.42)
Housewife	203 (98.5)	3 (1.5)	–	0.729^b^	1.19 (0.19, 9.11)	1.03 (0.17, 7.95)
Technician	362 (97.6)	9 (2.4)			2.00 (0.51, 13.22)	2.08 (0.50, 14.27)
Business	1,088 (97.6)	27 (2.4)			2.00 (0.59, 12.46)	1.99 (0.58, 12.50)
Others	107 (99.1)	1 (0.9)			0.75 (0.03, 7.95)	0.71 (0.03, 7.69)
**Air-conditioner use**						
No	165 (97.6)	4 (2.4)	–	0.786^b^	1.08 (0.32, 2.70)	1.08 (0.32, 2.81)
Yes	1,865 (97.8)	42 (2.2)			Reference	Reference
**Use of mosquito coils**						
No	1,830 (97.7)	43 (2.3)	–	0.618^b^	1.57 (0.56, 6.50)	1.65 (0.59, 6.93)
Yes	200 (98.5)	3 (1.5)			Reference	Reference
**Exercise**						
No	211 (95.9)	9 (4.1)	–	0.053^b^	2.10 (0.94, 4.22)	2.15 (0.95, 4.40)
Yes	1,819 (98.0)	37 (2.0)			Reference	Reference
**Vitamin consumption**						
**(times/week)**						
<5	742 (97.9)	16 (2.1)	–	0.228^b^	Reference	Reference
≥5	1,211 (97.9)	26 (2.1)			1.00 (0.54, 1.91)	1.03 (0.55, 1.99)
Refused to answer/Missing	77 (95.1)	4 (4.9)			2.41 (0.68, 6.76)	2.92 (0.70, 9.23)
**Vegetable consumption**						
**(times/week)**						
≤ 7	199 (98.5)	3 (1.5)	–	0.840^b^	Reference	Reference
8–14	718 (97.8)	16 (2.2)			1.48 (0.49, 6.40)	1.72 (0.55, 7.52)
≥15	1,074 (97.6)	26 (2.4)			1.61 (0.56, 6.78)	1.80 (0.62, 7.69)
Refused to answer/Missing	39 (97.5)	1 (2.5)			1.70 (0.08, 13.68)	0.82 (0.03, 8.88)

*BMI, body mass index*.

a *Variables in this table were simultaneously input into the logistic model*.

b *Fisher's exact test*.

The multivariate logistic regression analysis showed higher risks of dengue infection in pregnant women with age ≥35 years old (RR = 1.58, 95% CI: 0.85, 2.90), education ≤12 years (RR = 1.24, 95% CI: 0.63, 2.43), yearly income per capita <100,000 yuan (RR = 1.30, 95% CI: 0.67, 2.66), pre-pregnancy BMI <24.0 (RR = 1.47, 95% CI: 0.61, 4.35), no use of mosquito coils (RR = 1.65, 95% CI: 0.59, 6.93), and no exercise during pregnancy (RR = 2.15, 95% CI: 0.95, 4.40), compared with the corresponding reference group (Table 2).

### Association Between Maternal Fe Exposure Levels and Dengue IgG Antibody

**Figure 2:**
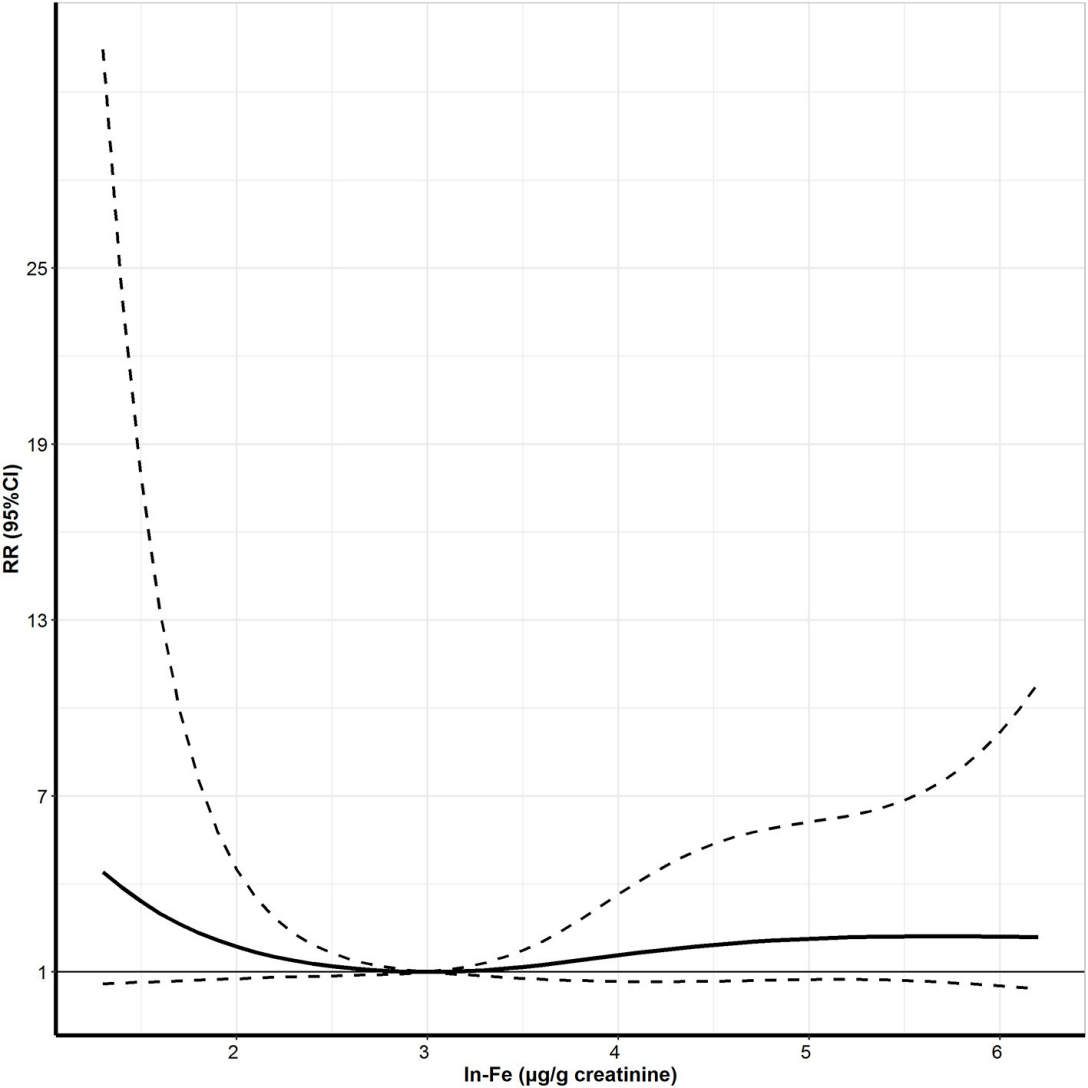
The exposure-response curve between urine Ln-Fe concentration and positive IgG antibody of DENV in pregnant women. Ln- Fe: the urinary Fe concentrations were corrected by creatinine and transformed by natural logarithm. Adjusted for maternal age, maternal education, maternal occupation, yearly income per capita, pre-pregnancy BMI, air-conditioner use, use of mosquito coils, exercise, vitamin consumption, and vegetable consumption.

Figure 2 shows the exposure-response curve between maternal urinary ln-Fe levels and dengue IgG antibody. We observed a U-shaped relationship between ln-Fe concentration and the risk of IgG antibody, with higher risk of positive IgG in women with low or high urinary ln-Fe concentrations. After dividing the ln-Fe concentrations into groups and compared with the second group (2.0–2.9 μg/g creatinine), the adjusted relative risks of IgG positive in the first (≤2.0 μg/g creatinine,), third (3.0–3.9 μg/g creatinine), fourth (4.0–4.9 μg/g creatinine) and fifth (≥5.0 μg/g creatinine) groups were 4.16 (95% CI: 0.78, 19.91), 1.93 (95% CI: 0.65, 7.08), 2.19 (95% CI: 0.65, 8.51), and 2.42 (95% CI: 0.46, 11.33), respectively. However, none of the associations was statistically significant (Table 3).

**Table 3:** Associations (RR, 95% CI) between urine Ln-Fe concentration and positive IgG antibody of DENV in pregnant women.

**ln-Fe (μg/g creatinine)^a^** **(*n* = 1,348)**	**IgG Negative** ***n* (%)**	**IgG Positive** ***n* (%)**	**χ^2^**	***P***	**Crude** **RR (95% CI)**	**Adjusted** **RR (95% CI)^b^**
Group1 (≤2.0)	59 (95.2)	3 (4.8)			4.41 (0.85, 20.50)	4.16 (0.78, 19.91)
Group 2 (2.0–2.9)	347 (98.9)	4 (1.1)			Reference	Reference
Group 3 (3.0–3.9)	503 (97.9)	11 (2.1)	–	0.320^c^	1.90 (0.64, 6.89)	1.93 (0.65, 7.08)
Group 4 (4.0–4.9)	279 (97.6)	7 (2.4)			2.18 (0.65, 8.38)	2.19 (0.65, 8.51)
Group 5 (≥5.0)	132 (97.8)	3 (2.2)			1.97 (0.38, 9.06)	2.42 (0.46, 11.33)

a *Ln- Fe: the urinary Fe concentrations were corrected by creatinine and transformed by natural logarithm*.

b *Adjusted for maternal age, maternal education, maternal occupation, yearly income per capita, pre-pregnancy BMI, air-conditioner use, use of mosquito coils, exercise, vitamin consumption, and vegetable consumption*.

c *Fisher's exact test*.

## Discussion

In this preliminary study, we observed that the seroprevalences of dengue virus infection among pregnant women in Guangzhou in 2016–2017 was close to the level in general population, and further found that higher or lower Fe exposure levels may increase the risk of dengue infection in pregnant women. These findings extended our understanding of the epidemic of dengue fever in pregnant women and its risk factors, which is of great significance for preventing dengue infection and the potential adverse consequences in pregnant women.

The positive prevalence of dengue IgG antibody was 2.22% in current study, which was much lower than that in pregnant women of some countries such as India (30.41%) (7) and Malaysia (32.4%) (8). This is probably since dengue is not an endemic disease in Guangdong Province, and imported cases are one of the main causes of local outbreaks of dengue fever in Guangdong. A previous study conducted by Liu et al. in Guangdong Province in 2016 reported that the seroprevalence of IgG in pregnant women was 3.15% (33), which is slightly higher than our finding. This difference may be related to the effect of dengue outbreak in 2014, during which an unprecedented outbreak of dengue occurred across Guangdong province, and total of 45,224 dengue fever cases were reported (28). It was suggested that the outbreak of dengue could substantially increase the prevalence of IgG antibody in population, and the IgG prevalence would gradually decrease after the outbreak (29, 32). For example, Guo et al. found that after the peak incidence of dengue fever in 2002, the prevalence of dengue IgG antibodies in healthy people gradually decreased from 4.81% in 2003 to about 1.20% in 2005 in Guangdong Province, China (29). Liu et al.'s study was conducted in 2016, two years after the outbreak of dengue in 2014. However, the present study was conducted during 2016–2017, which has a longer interval after the 2014 outbreak, which may partially explain the slightly lower prevalence of IgG antibody in this study. In contrast, the seroprevalence of dengue IgG antibody in the healthy population from Guangdong Province in 2012 was about 2.0%, which is consistent with our finding (32). This may indicate that the influence of dengue outbreak in 2014 on human's IgG antibody substantially declined during 2016–2017.

Our findings suggested that the risk of dengue infection may be related to several maternal demographic characteristics including age, occupation, education, and income level, which is consistent with previous studies (41–43). Pregnant women with an older age may be more likely exposed to dengue virus over time (41). The higher risk of dengue infection in manual workers may be due to their longer outdoor exposure, secreting a lot of sweat, which could increase their exposures to mosquitoes (44). In addition, pregnant women with lower education and less income levels may have worse living conditions than others, and lack knowledge about dengue fever prevention and control (45), thereby increasing the chance of being bitten by Aedes mosquitoes, which may increase their infection of dengue virus.

We also observed that the risk of dengue infection may be negatively associated with maternal behaviors including usage of air-conditioning, mosquito-repellent incense, and maternal exercise, which was consistent with previous studies (22, 46). Air conditioning usage could cool down the indoor environment, and the doors and windows were closed to reduce the chances of mosquitoes entering the room, thereby reduce the possibility of dengue infection (22). The indoor use of mosquito-repellent incense creates a relatively mosquito-free indoor environment, which reduces the chance of mosquito bites, and ultimately reduces the risk of DENV infection in pregnant women. In addition, it was suggested that exercise can directly regulate the immune system, such as macrophages, natural killer cells, and factors (47). Therefore, the regular and moderate exercise can enhance the physique and immunity of pregnant women, and hence reduce the risk of dengue fever infection. However, our multiple regression analysis showed that no statistically significant factors were found, one possible reason is that the sample size of this study is insufficient. But the results have significant implications, and further research is needed to confirm these results.

A U-shaped relationship was found between ln-Fe concentration and the risk of positive IgG antibodies. Higher risks of DENV infections were observed for pregnant women with lower or higher Fe concentrations, though the associations were not statistically significant. Although no study was found to investigate the relationship between the Fe concentration and dengue infection in pregnant women, some studies have reported associations of serum Fe concentration with infection of many viruses. Some studies found that the iron salt ferric ammonium citrate had a significant inhibitory effect on a variety of viruses, such as Influenza A virus (IAV), human immunodeficiency virus (HIV), Zika virus (ZIKV), and Enterovirus 71 (EV71) (48). Some studies had also found that iron metabolism affects the Hepatitis C virus (HCV) and HIV infection (49–51). An animal experiment has also reported that serum iron in human blood can affect mosquitoes infecting DENV (25). Mosquitoes can use serum iron to activate the activity of reactive oxygen species (ROS) in the intestinal epithelium, thereby inhibiting DENV infection (25).

The underlying mechanisms of the association between human iron levels and dengue infection is still unclear. In the present study, the greater risk of DENV infection with lower serum Fe concentration may be related to the level of ROS. It was reported that iron could activate the production of ROS by regulating heme, mediates the immune function of phagocytes, and inhibits dengue virus infection (52–54). As suggested by an animal experiment (25), the releases of ROS may inhibit the infection of dengue virus. In contrast, studies have also reported that low levels of ROS can promote virus replication, causing the host to be in a state of disease (55). However, as the iron concentrations exceed a threshold, the risk of DENV infection increased, which may be due to the excessive ROS production induced by the higher iron concentrations. The higher level of ROS may lead to severe oxidative stress, trigger inflammatory cytokine responses, and produce pathogenic-related proteins, which thereby cause cell damage, affect the immune response of human, increases the susceptibility to DENV infection (56, 57), and may even increase the risk of dengue hemorrhagic fever (DHF) (58–60). Our data also showed higher risks of pregnancy complications such as gestational hypertension, premature delivery, and low birth weight infants in pregnant women with higher Fe levels than women with median level of Fe (Supplementary Table 1), which further indicated the greater susceptibility in pregnant women with higher Fe concentration. Our findings suggest that the levels of iron and ROS in human body maybe be maintained within a certain range to reduce the risk of DENV infection. All these findings are needed to be verified in future studies with a larger sample size.

This study has several strengths. First, to our best knowledge, this is the first epidemiological study to investigate the relationship between maternal Fe exposure and risk of dengue fever infection. The results have extended our understanding of the infection of DENV in human body, and have important implications for the prevention of DENV infection. Second, we applied ELISA and ICP-MS methods to detect the maternal seroprevalence of DENV IgG antibody and Fe concentrations in maternal urines, respectively, which could provide a reliable exposure-response association between Fe concentration and DENV infection. Third, all participants were from a prospective birth cohort study, and the detailed individual information of study subjects were prospectively collected through face-to-face questionnaire interviews, which enables us to maximally reduce the recall bias and confounding bias.

This study also has several limitations. First, the study subjects were recruited from a single hospital, which may limit the generalization of our research results. Second, the sample size was small, and may have insufficient statistical power to determine the influence factors of dengue fever infection and the associations between Fe concentration and DENV infection risk. Third, a total of 728 participants did not donate urine samples, which may lead to a selection bias (Supplementary Table 2). Fourth, we measured maternal exposure to Fe concentration in urine only at one point of time, which does not reflect the long-term Fe level of pregnant women throughout the pregnancy. Fifth, we did not test the iron level in maternal blood samples, although the iron level in blood is more stable to represent the internal exposures than that in urine samples. In addition, the iron level in urine may also be vulnerable to factors such as diet. However, the individual level of iron in urine was adjusted for by urine creatinine, and maternal nutrition was adjusted for in the data analyses. Sixth, the neutralization test is a key laboratory method for detecting dengue infection, such as plaque reduction neutralization test (PRNT), which is generally regarded as the “gold standard” of the most accurate serological test for the diagnosis of DENV (61). However, in this study, we used the ELISA method, which differs in sensitivity and specificity from the neutralization test. Finally, the IgG antibodies are generally detectable after the first week of illness in serum and up to several months of infection and even could be present lifelong. However, the IgG antibody of pregnant women was detected only at the time of childbirth in this study, and we were not able to determine whether the infection of dengue virus occurred during the pregnancy. Therefore, the associations of DENV infections with maternal characteristics may be biased. Given the above limitations, we hope to conduct a multi-community-based cohort study in the future, using key laboratory methods such as PRNT to detect DENV IgG antibody levels in maternal blood samples, and to explore the relationship between maternal serum iron levels and dengue infection, to further expand our understanding of the relationship between maternal dengue fever infection and their iron levels.

In conclusion, the seroprevalence of dengue IgG antibody in pregnant women was comparable to the general population in Guangzhou, China. The risk of DENV infection may be associated with maternal demographic characteristics and behaviors. Both maternal low and high Fe concentrations may be positively associated with the risk of DENV infection in pregnant women. These findings can expand our understanding of risk of DENV infection, and help to prevent the DENV infection in pregnant women.

## Data Availability Statement

The raw data supporting the conclusions of this article will be made available by the authors, without undue reservation.

## Ethics Statement

The studies involving human participants were reviewed and approved by the Ethics Committee of the Review Board of Guangdong Provincial Center for Disease Control and Prevention. The patients/participants provided their written informed consent to participate in this study.

## Author Contributions

JW and JS wrote the manuscript. BZ and TL conceived and designed the study and reviewed and revised the manuscript. LS, YY, HC, JX, GH, JH, GC, HZ, XD, and WM contributed to data collection, statistical analysis, and visualization. All authors contributed to the article and approved the submitted version.

## Funding

This study was funded by the National Natural Science Foundation of China (42175181, 81874276, and 81773497); Natural Science Foundation of Guangdong Province (2019A1515011264); the Science and Technology Program of Guangzhou (202102080565 and 201707010037), and Open Project of Guangdong Provincial Key Laboratory of Tropical Disease Research.

## Conflict of Interest

The authors declare that the research was conducted in the absence of any commercial or financial relationships that could be construed as a potential conflict of interest.

## Publisher's Note

All claims expressed in this article are solely those of the authors and do not necessarily represent those of their affiliated organizations, or those of the publisher, the editors and the reviewers. Any product that may be evaluated in this article, or claim that may be made by its manufacturer, is not guaranteed or endorsed by the publisher.
