# Supplementary material for: The Seroprevalence of Dengue Virus Infection and Its Association With Iron (Fe) Level in Pregnant Women in Guangzhou, China
Source: Front Med (Lausanne). 2021 Dec 10;8:759728. doi: 10.3389/fmed.2021.759728 (PMC8702999; doi:10.3389/fmed.2021.759728)
Supplement: Supplementary file 1 [file Table_1.DOCX]

Supplementary Material

# Supplementary Tables

**Supplementary Table 1.** Incidence of pregnancy complications in pregnant women with different urine Fe concentrations.

| ln-Fe (μg/g creatinine)  (*n* = 1348) | N (%) | Gestational hypertension *n* (%) | Premature delivery *n* (%) | Low birth weight infant *n* (%) |
| --- | --- | --- | --- | --- |
| Group1 (≤2.0) | 62 (4.6) | 0 (0.0) | 1 (1.6) | 1 (1.6) |
| Group 2 (2.0~) | 351 (26.0) | 5 (1.4) | 4 (1.1) | 4 (1.1) |
| Group 3 (3.0~) | 514 (38.2) | 6 (1.2) | 4 (0.8) | 8 (1.6) |
| Group 4 (4.0~) | 286 (21.2) | 4 (1.4) | 4 (1.4) | 4 (1.4) |
| Group 5 (≥5.0) | 135 (10.0) | 5 (3.7) | 2 (1.5) | 4 (3.0) |

Ln-Fe: the urinary Fe concentrations were corrected by creatinine and transformed by natural logarithm.

**Supplementary Table 2.** General characteristics between pregnant women who provided urine samples or not.

|  | Urine samples | | $\chi$^2^ | *P* |
| --- | --- | --- | --- | --- |
|  | Yes *n* (%) | No *n* (%) |  |  |
| Maternal age (years) |  |  | 0.02 | 0.877 |
| <35 | 928 (68.8) | 498 (68.4) |  |  |
| ≥35 | 420 (31.2) | 230 (31.6) |  |  |
| Maternal education (years) |  |  | 0.42 | 0.515 |
| ≤12 | 549 (40.7) | 308 (42.3) |  |  |
| >12 | 799 (59.3) | 420 (57.7) |  |  |
| Yearly income per capita  (×1,000 Yuan) |  |  | 8.42 | 0.015 |
| <100 | 852 (63.2) | 503 (69.1) |  |  |
| ≥100 | 482 (35.8) | 215 (29.5) |  |  |
| Refused to answer/Missing | 14 (1.0) | 10 (1.4) |  |  |
| Pre-pregnancy BMI (kg/m^2^) |  |  | 0.68 | 0.411 |
| <24.0 | 1176 (87.2) | 625 (85.9) |  |  |
| ≥24.0 (Overweight or Obesity) | 172 (12.8) | 103 (14.1) |  |  |
| Maternal occupation |  |  | 14.14 | 0.015 |
| Unemployment | 70 (5.2) | 43 (5.9) |  |  |
| Manual worker | 138 (10.2) | 68 (9.3) |  |  |
| Housewife | 265 (19.7) | 106 (14.6) |  |  |
| Technician | 707 (52.4) | 408 (56.1) |  |  |
| Business | 109 (8.1) | 54 (7.4) |  |  |
| Others | 59 (4.4) | 49 (6.7) |  |  |
| Air-conditioner use |  |  | 0.09 | 0.767 |
| No | 112 (8.3) | 57 (7.8) |  |  |
| Yes | 1236 (91.7) | 671 (92.2) |  |  |
| Use of mosquito coils |  |  | 2.08 | 0.149 |
| No | 1226 (90.9) | 647 (88.9) |  |  |
| Yes | 122 (9.1) | 81 (11.1) |  |  |
| Exercise |  |  | 10.18 | 0.001 |
| No | 121 (9.0) | 99 (13.6) |  |  |
| Yes | 1227 (91.0) | 629 (86.4) |  |  |
| Vitamin consumption (times/week) |  |  | 0.88 | 0.645 |
| <5 | 502 (37.2) | 256 (35.2) |  |  |
| ≥5 | 794 (58.9) | 443 (60.8) |  |  |
| Refused to answer/Missing | 52 (3.9) | 29 (4.0) |  |  |
| Vegetable consumption (times/week) |  |  | 14.82 | 0.002 |
| ≤7 | 110 (8.2) | 92 (12.6) |  |  |
| 8~14 | 505 (37.5) | 229 (31.5) |  |  |
| ≥15 | 708 (52.4) | 392 (53.8) |  |  |
| Refused to answer/Missing | 25 (1.9) | 15 (2.1) |  |  |

BMI: body mass index.
